# Human Prostate Side Population Cells Demonstrate Stem Cell Properties in Recombination with Urogenital Sinus Mesenchyme

**DOI:** 10.1371/journal.pone.0055062

**Published:** 2013-01-31

**Authors:** Barbara A. Foster, Kalyan J. Gangavarapu, Grinu Mathew, Gissou Azabdaftari, Carl D. Morrison, Austin Miller, Wendy J. Huss

**Affiliations:** 1 Department of Pharmacology and Therapeutics, Roswell Park Cancer Institute, Buffalo, New York, United States of America; 2 Medical and Molecular Genetics, Indiana University School of Medicine, IUPUI, Indianapolis, Indiana, United States of America; 3 Department of Pathology, Roswell Park Cancer Institute, Buffalo, New York, United States of America; 4 Department of Biostatistics, Roswell Park Cancer Institute, Buffalo, New York, United States of America; 5 Department of Urologic Oncology, Roswell Park Cancer Institute, Buffalo, New York, United States of America; Institut de Génomique Fonctionnelle de Lyon, France

## Abstract

Stem cell enrichment provides a tool to examine prostate stem cells obtained from benign and malignant tissue. Functional assays can enrich stem cells based on common stem cell phenotypes, such as high ATP binding cassette (ABC) transporter mediated efflux of Hoechst substrates (side population assay). This functional assay is based upon mechanisms that protect cells from environmental insult thus contributing to the survival and protection of the stem cell population. We have isolated and analyzed cells digested from twelve clinical prostate specimens based on the side population assay. Prostate stem cell properties of the isolated cells were tested by serial recombination with rat urogenital mesenchyme. Recombinants with side population cells demonstrate an increase in the frequency of human ductal growth and the number of glands per recombinant when compared to recombinants with non-side population cells. Isolated cells were capable of prostatic growth for up to three generations in the recombination assay with as little as 125 sorted prostate cells. The ability to reproducibly use cells isolated by fluorescence activated cell sorting from human prostate tissue is an essential step to a better understanding of human prostate stem cell biology. ABC transporter G2 (ABCG2) was expressed in recombinants from side population cells indicating the side population cells have self-renewal properties. Epithelial cell differentiation of recombinants was determined by immunohistochemical analysis for expression of the basal, luminal, and neuroendocrine markers, p63, androgen receptor, prostate specific antigen, and chromogranin A, respectively. Thus, the ABCG2 expressing side population demonstrates multipotency and self-renewal properties indicating stem cells are within this population.

## Introduction

Prostate epithelial stem cells are defined as possessing the capability to generate prostatic epithelium through the properties of self-renewal and multipotency. These essential features of prostate stem cells can be tested *in vivo* in the tissue recombination assay. Recombination of an epithelial stem cell with mesenchyme derived from embryonic urogenital sinus mesenchyme (UGM) and grafting the recombinant under the renal capsule of an immune compromised host animal re-establishes the stem cell niche and allows for the dynamic assaying of stem cell properties within an *in vivo* system. The classic application of urogenital tissue recombination technology was the demonstration that heterospecific (between species) recombinations of UGM induced differentiation and branching morphogenesis in transplanted epithelium from different species [1]. The recombined mesenchymal/stromal environment has profound effects on the phenotype of the associated epithelium. Studies using adult human prostate epithelium in tissue recombination assays demonstrate that the stem cells in the prostate epithelial compartment can respond to the inductive effect of rodent UGM by committing to proliferation, undergo branching morphogenesis and differentiation [2]. In addition, the human prostatic epithelium dictates smooth muscle differentiation in the rat UGM (rUGM), inducing the appearance of thick sheets of smooth muscle characteristic of human, not rat, prostate [2]. Tissue recombination has been used to demonstrate that the mouse prostate stem cell is located in the proximal region of the prostatic duct, and can be enriched by isolating Sca expressing cells [3], [4]. Furthermore, single mouse prostate cells expressing Lin^−^Sca^+^CD133^+^CD44^+^CD117^+^ generated prostate tissue for one generation when recombined with rUGM at a low frequency [5]. Recent studies using lineage-tracing methods in prostate regeneration suggest that basal and luminal stem cells repopulate the respective compartments [6].

Tissue recombination assays have demonstrated the presence of stem cells in prostaspheres generated from human specimens [7], primary cells grown from human prostate specimens [8], and spontaneously immortalized human prostate cell lines [9]. There have been few studies using tissue recombination to test stem cell properties of cells isolated from human prostate tissue based on the expression of putative stem cell markers. Recently, tissue recombination of human prostate cells isolated from tissue based upon Epcam, CD44, and CD49f expression in recombination with human fetal stromal cells induced sphere forming capabilities in Epcam^+^CD44^+^CD49f^hi^ expressing cells compared to tubule forming cells that were Epcam^+^CD44^−^CD49^hi^ [10]. However these types of analysis use ∼10^5^ cells and are prohibitive when studying very rare population of cells in limited amounts of tissue. Additionally, the lack of a reliable technique to isolate sufficient numbers of putative stem cells from human prostate tissue has limited the testing of prostate stem cell properties using tissue recombination assays [7], [8].

We and others have isolated and validated a potential stem cell population, the side population, from clinical prostate tissue [11], [12], [13], [14]. The side population assay is a functional assay first used to enrich for hematopoietic stem cells. The cells are selected with fluorescence activated cell sorting (FACS) technique based upon the efflux of Hoechst 33342 dye mediated by the presence of functional ABC transporters [15]. Several ABC transporters can contribute to defining the side population, but ABCB1 (formally known as Mdr1 and p-glycoprotein) and ABCG2 (formally known as Bcrp) are the main contributors [16]. ABCG2-mediated efflux of Hoechst is the molecular determinant of the side population phenotype with stem cell properties in the mouse bone marrow as demonstrated in Abcg2 and Abcb1a/b deficient mice [17], [18]. Traditionally the efflux of Hoechst 33342 from cells is used to identify the stem cell side population in flow histograms, we have recently switched to a less cytotoxic fluorescent substrate of ABCG2, DyeCycle Violet (DCV) [19]. To ensure that DCV efflux was mediated by ABCG2, the protocol was validated using mouse bone marrow aspirates and a specific inhibitor of ABCG2 function, fumitremorgin C (FTC), [11]. We utilized the DCV protocol to detect the side population in several human prostate cell lines and clinical prostate specimens [11].

The human prostate side population has some stem cell like characteristics, but has not yet been tested in the tissue recombination assay for tissue regeneration. The side population from benign prostate specimens expresses high levels of CD133, p21, p27, Msi, cytokeratin 5, and cytokeratin 14 and low levels of p63 and prostate specific antigen (PSA) compared to non-side population cells [13]. Side and non-side population cells isolated from benign and malignant human tissues form spheres with no statistical difference in sphere forming efficiency between side and non-side population or between benign and malignant tissue [13]. An affymetrix array analysis of the prostate side population and cells expressing ABCG2 (isolated by magnetic beads conjugated to an antibody against ABCG2) isolated from clinical prostate specimens indicates that there is considerable overlap of gene expression between the side population and ABCG2 expressing cells from the prostate [20]. The ABC transporters, ABCG1, ABCG2, ABCB1, and ABCE1, were expressed at higher levels in the side population compared to ABCG2 expressing cells isolated with magnetic beads [13], [20]. Also, the inhibition of ABCG2 in prostate cells led to increased expression of nuclear androgen receptor (AR) and increased the intracellular level of dihydrotestosterone (DHT) [21]. Thus, ABCG2 function may play a key role in the maintenance of the prostate stem cell phenotype.

The previous results demonstrating that the side population enriches for stem cells in other tissues and the ability to isolate a consistent number of side population cells from human prostate tissue led to these investigations. The current study determines if the side population from freshly digested prostate tissue is enriched for prostate stem cells, as demonstrated by measuring the ability to generate differentiated human ductal growth in serial recombination with rUGM.

## Materials and Methods

### Ethics Statement

All of the tissue samples were collected under an Institutional Review Board (IRB)-approved protocol at Roswell Park Cancer Institute (RPCI). Specimens were collected after IRB-approved written consent from the patient was obtained at RPCI. All experiments were conducted and approved under our Institutional Animal Care and Use Committee (IACUC) at RPCI under protocol ID number 1201 M.

### Cell Isolation and Gating Strategy for Side Population Assay

Fresh human benign prostate tissue and prostate cancer tissue, harvested from radical prostatectomy and cystoprostatectomy surgical specimens stored in static preservation solution (SPS-1™) (Organ Recovery Systems) (4°C) was obtained from the Pathology Resource Network at RPCI. Areas of radical prostatectomy tissue specimens that were greater than 90% cancer were identified by analysis of frozen sections of adjacent tissue by a pathologist prior to distribution of the specimen [22]. Enzymatic tissue digestion and single cell suspension was prepared as described [23].

Cells isolated from prostate tissue were labeled with DCV (Invitrogen) according to a protocol modified from Telford *et al*. [19] as previously described [11], [23]. The gating strategy for sorting the side and non-side populations were performed as previously described [11], [23] adhering to the protocol suggestion proposed [24]. Cells were sorted into 0.5 ml of Hank’s buffer (Invitrogen) +5% FBS (Gibco) kept at 4°C and used within 30–90 minutes of sorting for recombination experiments.

### Tissue Recombination

rUGMs from embryonic day 18 Fisher/344 rats (Harlan) were dissected as described [25]. The rUGMs were incubated on a 1.5% agar plate at 37°C until the sorted cell population or epithelial tissue from previous generation recombinant was added ∼ 0–180 minutes. First generation recombinants were generated by aliquoting 5.0×10^1^–2×10^3^ side or 1.25×10^2^–5.0×10^4^ non-side population sorted cells in 20 µL Hanks’ buffer +5% FBS isolated from human prostate tissue specimens with a single rUGM or alone in a 200 µL microcentrifuge tube. Cells were spun onto the rUGM or pelleted alone at 1588 g for 10 min at 4°C and the media was removed leaving the meniscus layer. Tubes containing cells plus rUGM were incubated overnight at 37°C in an atmosphere of 5% CO_2_. Cells alone, without rUGM, were resuspended in 4 µL neutralized collagen solution and incubated overnight at 37°C in an atmosphere of 5% CO_2_ on an agar plate. Cells plus rUGM were placed into 4 µL collagen solution the next morning and allowed to solidify at 37°C in an atmosphere of 5% CO_2_ for 60–90 minutes prior to engraftment. Up to three rUGMs without sorted cells were grafted in most experiments as a negative control. Collagen solution is comprised of 10 mg/mL type I rat tail collagen (Roche) dissolved in 0.2% acetic acid and neutralized with a solution comprised of: Earle’s Balanced Salt Solution (Sigma) 6.6×; 200 mM NaHCO_3_; 50 mM NaOH, immediately prior to use.

### Renal Grafting

**Figure 1:**
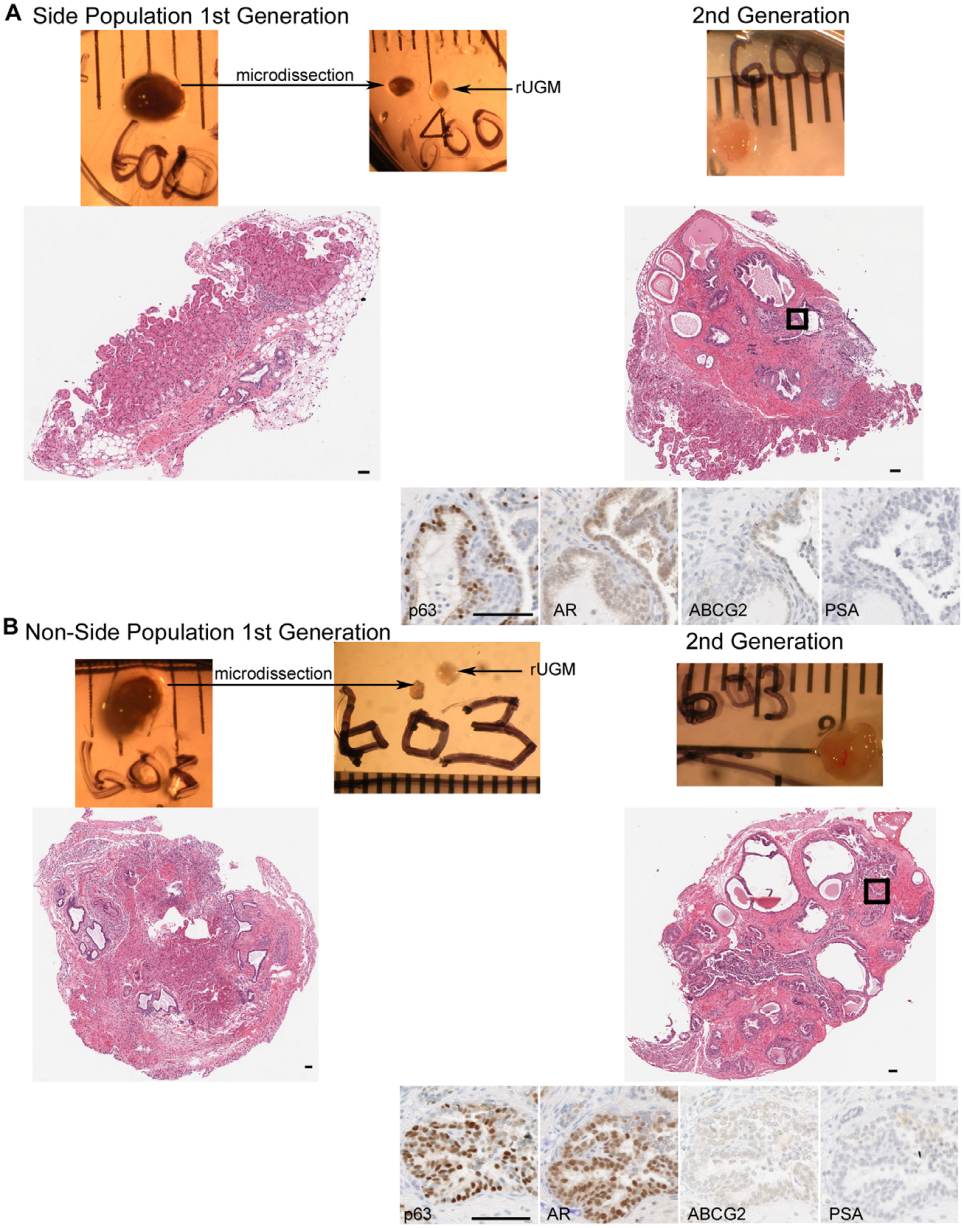
Serial Recombination with new rUGM. A) First generation recombinant (H&E staining) generated with 1000 side population cells isolated from 9NT, micro-dissected piece of the recombinant and new rUGM used for serial recombination, and second generation growth (H&E staining and IHC analysis). B) First generation recombinant (H&E staining) generated with 400 non-side population cells isolated from 9NT, micro-dissected piece of the recombinant and new rUGM used for serial recombination, and second generation growth (H&E staining and IHC analysis). Ruler scale = mm; Scale bar = 50 µm.

Cells plus rUGM or cells alone in collagen were grafted under the renal capsule in host mice as described [26]. Twelve-week old CBySmn.CB 17 Prkdcscid (SCID) male host mice were castrated and implanted subcutaneously with 12.5 mg sustained-release testosterone pellets (Innovative Research of America) for 48 hours to establish serum testosterone levels at ∼4 ng/ml prior to implantation of 1–4 grafts under each renal capsule. Following 4–11 weeks of growth, grafts were harvested, weighed, photographed, and when ductal growth was present, a ∼1 mm cube of tissue containing both epithelial and stromal compartment was micro-dissected from the graft and used for serial recombination with new rUGM (Figure 1). If multiple replicates demonstrated ductal growth from side and non-side population groups, one of each group was selected by investigator (B.A.F.) blind to the group and was serially recombined. The remaining portion of the recombinant was fixed in 10% neutral buffered formalin (VWR), for 24 hours, dehydrated, paraffin embedded, sectioned at 5 µm placed on ProbeOn-Plus slides (Fisher), and stained with hematoxylin and eosin (H&E).

### Immunohistochemistry (IHC)

Slides were deparaffinized in xylene, rehydrated through a graded series of alcohol washes, and equilibrated in PBS. Antigen retrieval was performed in 10 mM citric acid pH 6.0 for 30 minutes in a steamer. Slides were incubated with appropriate primary antibodies diluted in PBS (Invitrogen) with 5% goat serum (Vector): 1∶50 dilution of mouse monoclonal anti-p63 clone 4A4 (Santa Cruz, sc-8431); 1∶100 dilution of mouse monoclonal anti-PSA clone ER-PR8 (Dako, M0750); 1∶80 dilution of mouse monoclonal anti-ABCG2 clone 5D3 (e-Biosciences, 12-8888-71); 1∶80 dilution rabbit polyclonal anti-AR clone PG-21 (Millipore, 06-680); 1∶600 dilution rabbit polyclonal anti-chromogranin A (Dako, A0430); or 1∶100 rabbit monoclonal anti-AMACR (Dako, M3616) for 30 minutes at 37°C. All slides were incubated with the appropriate biotinylated secondary antibody diluted in PBS with 5% goat serum: 1∶1000 dilution goat anti-mouse IgG (Vector Laboratories Inc., BA9200) or 1∶1000 dilution goat anti-rabbit IgG (Vector Laboratories Inc., BA1000) for 20 minutes at 37°C. Immunoreactive antigens were detected using Strepavidin (Vector Laboratories Inc., SA5704) and DAB. Human prostate tissue served as a positive control for p63, AR, PSA, chromogranin A, ABCG2, and Alpha-methylacyl-CoA racemace (AMACR) expression and no primary antibody controls served as negative controls for each experiment (Figure S1).

### Aperio Imaging Recombinant Histology and IHC

All slides containing H&E stained and immunostained recombinants were scanned on the ScanScope XT System in the Pathology Resource Network at RPCI and Images were analyzed and acquired with Spectrum Version 10.2.2.2317.

### Identification of Epithelial Origin by Fluorescence *In Situ* Hybridization (FISH) and Hoechst Staining

All recombinant tissue was stained with Hoechst to detect host mouse cell contamination as demonstrated by punctuate staining of mouse nuclei [27]. Slides were deparraffinized and coversliped with Vectashield containing 20 µg/mL Hoechst 33342. All experiments included mouse prostate tissue as a positive control (Figure S4C) and human prostate tissue as a negative control (Figure S4F).

Telomere FISH analysis was performed in order to identify the cells contributing to the development of prostate glands from the rUGM recombinants. Mammalian telomeres were detected as described [28]. The slides were visualized using an Olympus BX40 Olympus BX illuminator with appropriate filters. All FISH experiments included mouse prostate as a positive control (Figure S4A) and human prostate as a negative control (Figure S4D).

The number of glands was determined in all recombinants with human epithelial cells. When possible the number of glands with a continuous luminal epithelial layer was determined in two H&E stained slides, sectioned 50 µm apart (Slides 1 & 11 from serial sections). The average number of glands/recombinant was compared between the two sorted populations by Exact Wilcoxon test. When the specimen was not large enough to generate 11 sections, the number of glands were only determined in one section. Recombinants with no glands or only rodent epithelial glands were not included in the analysis.

### Statistical Analysis

Fisher’s exact test was performed with GraphPad Prism version 5.01 software and the Exact Wilcoxon test and Kaplan-Meier analysis was performed with SAS v9.3 software. The recombinant survival rate was defined as the proportion of cells demonstrating growth at each generation analyzed. The tabulated values show the number of cells in the risk set at the beginning of each cycle. Recombinants with growth in the third generation were censored. Kaplan-Meier methods provide a visual comparison of these survival distributions for the side and non-side recombinants. The null hypothesis of no difference in the survival distributions was assessed using the Log Rank test.

## Results

### Side and Non-Side Population Isolation from Clinical Prostate Specimens

**Figure 2:**
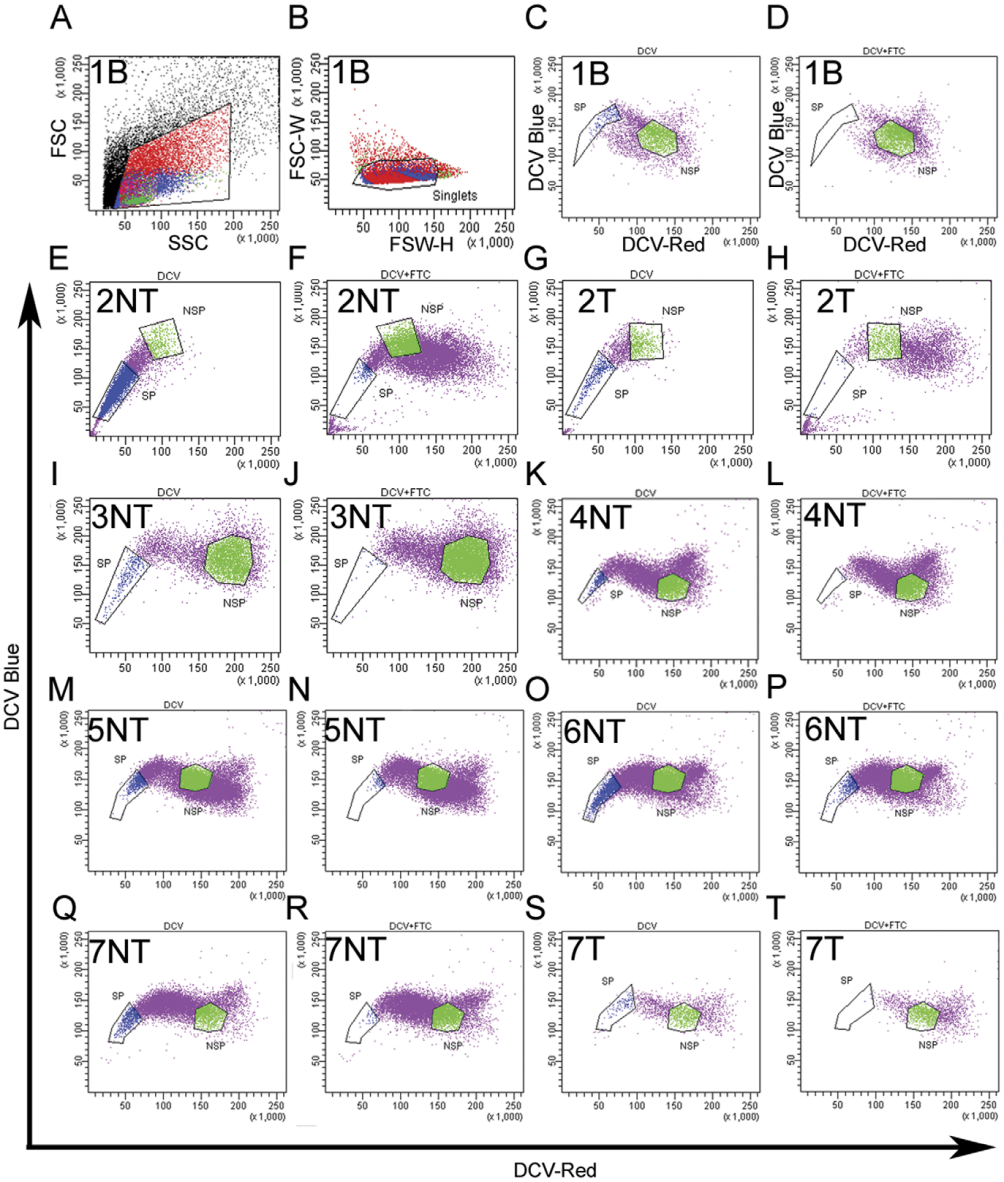
FACS isolation of the side population in human clinical specimens based upon DCV efflux. A) Live cells are identified and gated based on 7AAD staining and a plot of forward scatter (FSC) vs. side scatter (SSC). B) Cells gated in (A) are plotted and gated as viable singlets based on FSC-Width (FSC-W) vs. FSC-Height (FSC-H). C,E,G,I,K,M,O,Q,S) The gated viable singlets (B) are plotted in a double scatter plot between red (650 nm) and blue (450/40 nm) emission and are isolated based upon efflux of DCV. D,F,H,J,L,N,P,R,T) ABCG2-mediated DCV efflux is inhibited in the presence of FTC to establish where the side population (SP) and non-side population (NSP) gates should be placed. Purple: viable single cells; Green: NSP; Blue: SP.

Fresh prostate tissue was obtained from human prostate surgical specimens harvested from patients undergoing radical prostatectomy (11) and cystoprostatectomy (1) (Table 1). According to the method of Morrison *et al*, eleven radical prostatectomy specimens were procured, four of which contained areas of un-involved (non-tumor [NT]) and tumor (T) tissue while seven contained only non-tumor tissue [22]. A single benign (B) prostate specimen was procured from a cystoprostatectomy surgery performed to treat bladder cancer. The weights of procured/digested tissue, Gleason Grade, cell viability, percent of side and non-side population gated are as listed in Table 1. The Gleason Grades for the initial radical prostatectomy specimens were: 3+3, 3+4, and 4+3 (Table 1), the procured tumor tissue represents non-tumor areas or tumor areas surrounded by the graded specimen. Tissue was digested overnight and single viable cells were assayed for the side population phenotype. The side population was gated following gating out debris (Figure 2A) and single cell identification. Discrimination of viable and dead cells was performed with 7-AAD cell viability dye (Figure 2B). ABCG2 enriched side population cells were determined by gating the population of cells that were restricted from DCV efflux due to the inhibition of transporter activity in the presence of ABCG2 inhibitor FTC (Figure 2 and Figure S2). The gate placement recovered a relatively small number of cells ranging from 0.1–8.1% in non-tumor specimens (Figure 2 and Figure S2∶2NT-9NT, 11NT), 0.1–0.4% in tumor specimens (Figure 2; 2T and 7T and Figure S2∶10T & 12T) and 0.7% in the benign specimen (Figure 2B1) (Table 1). The flow cytographs in Figure 2 and Figure S2 represent side population in prostate specimens: distinct side populations were observed in all specimens and the non-side population gate was set by establishing a clear distinction from the side population gate.

**Table 1:** The percentage of total side population (SP) and non-side population (NSP) gated relative to total viable cells in tissue specimens from human benign prostate and prostate cancer specimens.

Specimen	Gleason Grade	Specimen Weight (g)	# Viable Cells	% Viable Cells	% SP Gated	% NSP Gated
1B	NA	4.000	3.70×10^4^	18.5	0.7	6.1
2NT	3+4	19.47	7.70×10^5^	13.1	8.1	0.4
2T		0.4	3.50×10^5^	11.7	0.4	0.5
3NT	3+3	2.390	5.90×10^5^	23.3	0.5	6.3
4NT	4+3	5.440	5.70×10^5^	10.7	0.1	0.8
5NT	3+3	5.430	1.10×10^5^	12.2	0.4	3.1
6NT	3+4	3.700	1.21×10^6^	27.0	0.8	2.5
7NT	3+4	7.580	7.70×10^5^	18.7	0.3	0.6
7T		0.270	3.90×10^5^	20.3	0.1	0.6
8NT	3+3	8.274	2.16×10^6^	37.6	0.2	6.6
9NT	3+3	4.764	5.80×10^5^	47.0	0.8	6.6
10NT	4+3	2.480	7.60×10^5^	44.1	0.2	6.4
10T		2.840	8.10×10^5^	46.9	0.3	3.6
11NT	4+3	3.880	1.50×10^6^	38.1	0.3	2.9
12NT	4+3	2.693	8.90×10^5^	67.5	0.4	4.7
12T		1.715	1.40×10^5^	68.8	0.4	4.4

NT: Histologically non-tumor areas from radical prostatectomies. T: Histologically tumor areas from radical prostatectomies. B: Benign prostate specimens from cystoprostatectomies.

### Serial Generation of Prostatic Tissue from Human Epithelial Cells Recombined with Inductive rUGM

To functionally test for stem cell properties, side and non-side population cells were recombined with inductive rUGM. Stem cells combined with rUGM in the recombination assay are capable of generating all the cell types of a prostate gland, hence demonstrating multipotency. In this study, equal and representative numbers of cells were sorted for the side population or non-side population phenotype and used in recombination with rUGM or suspended in collagen alone without rUGM, and implanted under the renal capsule of immune compromised host mice (Tables 2 and 3).

**Table 2:** Incidence of human ductal growth in recombinants with equal number of sorted cells.

		Side Population			Non-Side Population		
		Analyzed^(Growth)^			Analyzed^(Growth)^		
		Generation			Generation		
	# of Cells/Recombinant	1^st^	2^nd^	3^rd^	1^st^	2^nd^	3^rd^
1B	125	4^(1)^	1^(0)^		6^(1)^	1^(0)^	
1B	250	4^(2)^	2^(0)^		3^(0)^		
1B	2000				2^(0)^		
2NT	500	2^(1)^	1^(1)^	1^(1)^	2^(0)^		
2NT	1000	2^(0)^			2^(0)^		
2NT	2000	2^(0)^			2^(0)^		
2T	500	*			2^(0)^		
2T	1000	2^(0)^			2^(0)^		
2T	2000	2^(1)^	2^(1)^	1^(0)^	*		
3NT	250	3^(1)^	1^(1)^	1^(1)^	3^(0)^		
3NT	1000	3^(0)^			3^(0)^		
4NT	125	4^(4)^	3^(3)^	3^(3)^	3^(0)^		
4NT	250	3^(0)^			3^(1)^	1^(1)^	1^(1)^
4NT	500	3^(1)^	2^(1)^	1^(1)^	3^(2)^	1^(0)^	
5NT	125	3^(2)^	1^(0)^		2^(0)^		
5NT	500	2^(0)^			2^(0)^		
6NT	250	2^(1)^	ND		2^(0)^		
6NT	1000	2^(1)^	ND		2^(0)^		
7NT	500	2^(1)^	2^(0)^		2^(0)^		
7NT	2000	2^(0)^			2^(0)^		
7T	250	2^(0)^			2^(1)^	1^(1)^	ND
7T	500	2^(0)^			2^(1)^	2^(1)^	1^(0)^

* Grafts not analyzed due to host animal death. ND: Not Determined because the recombinant did not demonstrate visible ductal growth upon micro-dissection.

**Table 3:** Incidence of human ductal growth in recombinants with representative number of sorted cells.

		Side Population				Non-Side Population		
		Analyzed^(Growth)^				Analyzed^(Growth)^		
		Generation				Generation		
	# of Cells/Recombinant	1^st^	2^nd^	3^rd^	# of Cells/Recombinant	1^st^	2^nd^	3^rd^
8NT**	125	2^(1)^	2^(1)^	2^(1)^	3125	2^(0)^		
8NT	500	2^(1)^	ND		12500	2^(0)^		
8NT	2000	2^(0)^			50000	2^(0)^		
9NT	50	3^(0)^			400	3^(2)^	3^(1)^	1^(0)^
9NT	250	2^(0)^			2000	*		
9NT	1000	2^(2)^	2^(2)^	2^(0)^	8000	2^(0)^		
10NT	50	3^(0)^			1600	*		
10NT	250	1^(0)^ *			8000	2^(1)^ *	1^(1)^	ND
10T	50	3^(0)^			550	3^(0)^		
10T	250	3^(0)^			2750	3^(0)^		
10T	500	3^(1)^	2^(1)^	1^(1)^	5500	3^(1)^	2^(0)^	
10T					21507	4^(0)^		
11NT	100	*			1033	3^(0)^	*	
11NT	500	4^(0)^			5166	4^(1)^	*	
12NT	100	3^(0)^			1200	3^(1)^	2^(1)^	*
12NT	500	3^(0)^			6000	3^(1)^	1^(0)^	
12T	50	3^(1)^	2^(0)^		500	3^(1)^	2^(0)^	
12T	100	3^(3)^	1^(0)^		1000	3^(0)^		

* Grafts not analyzed due to host animal death. ND: Not Determined because the recombinant did not demonstrate visible ductual growth upon micro-dissection.

** There was a 33-fold ratio between non-side and side population cells isolated, however due to the number of cells isolated only a 25-fold ratio was possible for the recombinants from this specimen.

Upon harvest, tissue recombinants were examined for evidence of ductal growth under the dissecting microscope (Figure 1 and Figure S3). Cells suspended in collagen without rUGM did not grow. In the event of ductal growth, a small portion of epithelial tissue from the recombinant was micro-dissected, recombined with new rUGM (Figure 1), and implanted under the renal capsule of host mice. This step is critical for examining the capability of the transplanted human cells for continuous prostate epithelial generation, therefore defining the population of cells as manifesting multiple regeneration potential. At the time of harvest recombinants were processed for histology only (no serial recombination with rUGM), if there was no evidence of ductal growth or the ductal growth was obviously rodent was visible under the dissecting microscope (Figure S3) the recombinant was processed for histological analysis of microscopic ductal growth. This led to a selection procedure which resulted in some recombinants with histological growth not being serially passed and on the contrary some recombinants were serially passed although there was no histological evidence of human epithelial cell growth. Some grafted recombinants were not harvested due to scar tissue in the renal capsule but were counted as no growth. Recombinants not analyzed due to the death of host animal are noted in Tables 2 and 3.

**Figure 3:**
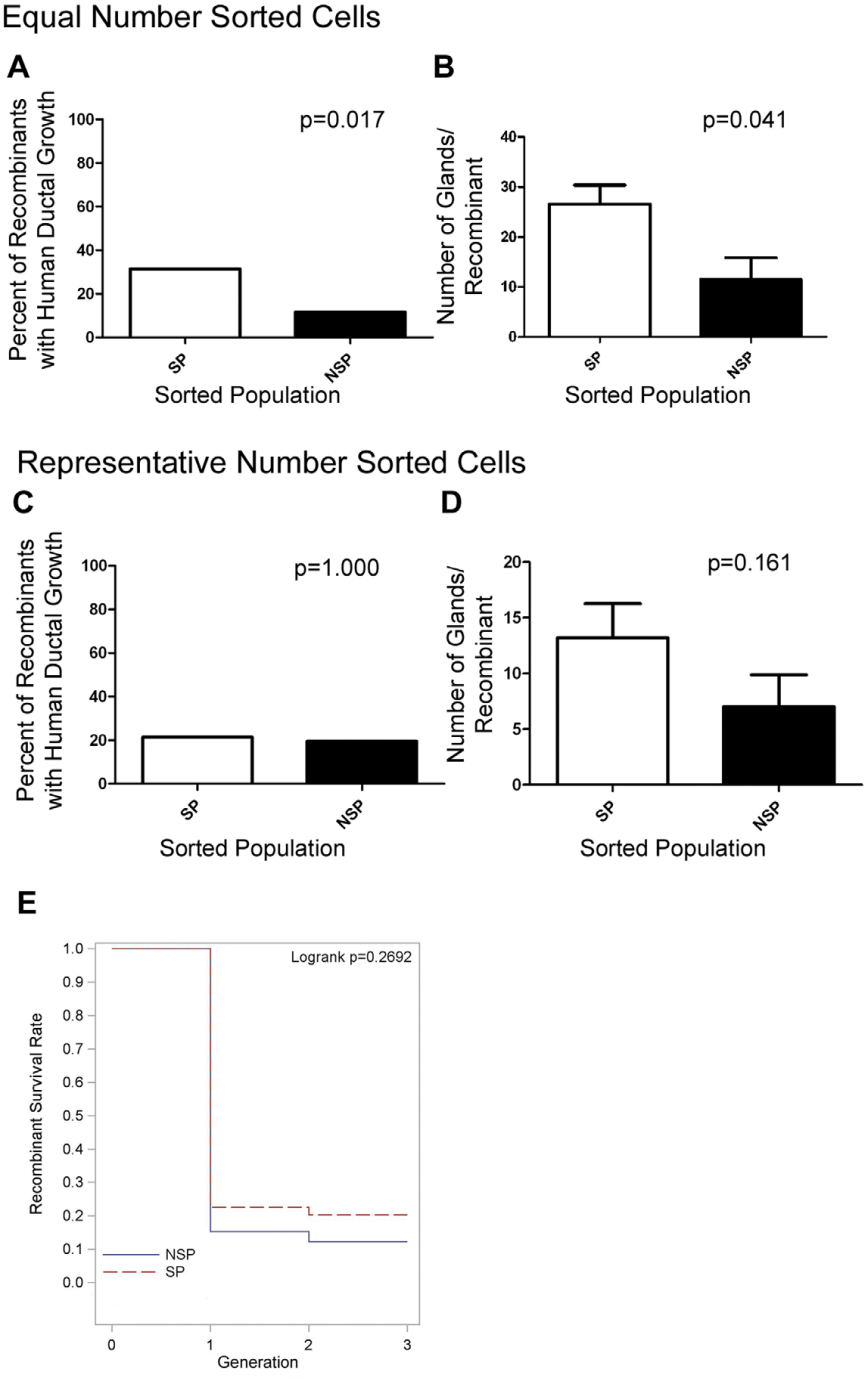
Ductal Growth of Recombinants from Side and Non-Side Population Cells. A) The percentage of recombinants with human ductal growth in the first generation with equal number of sorted cells side population n = 51 and non-sided population n = 52, analyzed by Fisher’s Exact two-sided test p = 0.017. B) The number of glands in recombinants with equal number of sorted cells derived from side population n = 19 and non-side population n = 6 was quantitated and analyzed by Exact Wilcoxon test p = 0.041. C) The percentage of recombinants with human ductal growth in the first generation with representative number of cells side population n = 42 and non-side population n = 45, analyzed by Fisher’s Exact two-sided test p = 1.000. D) The number of glands in recombinants with representative number of cells derived from side population n = 10 and non-side population n = 9 was quantitated and analyzed by Exact Wilcoxon test p = 0.161. E) Kaplan-Meier plot of recombinant survival rate in multiple generations, Logrank test p = 0.2692.

All recombinants were H&E stained to detect epithelial growth (Figure 1). The recombinants were assayed for infiltrating host mouse cells by staining the nuclei with Hoechst. Rodent telomeres were identified by FISH analysis to disqualify any glands with mouse or rat epithelial cells (Figure S4). Each recombinant was evaluated by W.J.H. who was blinded to contributing sorted population. First generation growth analysis was performed on 103 recombinants with equal number of side and non-side population sorted cells (Table 2). The recombinants were scored as containing human ductal growth or no human ductal growth (Figure 3A). The percent of recombinants with human ductal growth was more when using the side population (31%) compared to non-side population (12%) p = 0.017 (Figure 3A). The number of human glands was quantitated for each recombinant (Figure 3B). More glands formed in the recombinants derived from the side population than the non-side population (p = 0.041). Of the 103 first generation recombinants analyzed 44% did not demonstrate any ductal growth and 37% demonstrated some rodent ductal growth with or without human ductal growth.

First generation growth analysis was performed on 87 recombinants with representative number of sorted cells (Tables 1 and 3). In the viable cells recovered more non-side population cells were recovered than side population cells. We fixed the ratio of side to non-side population cells for each specimen based on the ratio recovered for each. Recombinations were performed with increasing number of cells, but the ratio of side to non-side population was fixed. For example, in specimen 12T, 0.44% of the viable cells were side population cells, while 4.4% were non-side population cells. Recombinations were performed with 50 side population, 500 non-side population, 100 side population, or 1000 non-side population cells and the resulting ductal growth was evaluated. There was no difference in the percentage of recombinants with human ductal growth (Figure 3C) or in the number of glands/recombinant (Figure 3D) between recombinants with representative number of side or non-side population cells.

The ability of the two cell populations to generate serial recombinant growth was compared by Kaplan-Meier methods and was not statistically significant p = 0.2692 (Figure 3E). The recombinant survival rate was calculated based on the number of recombinants grafted and eliminated recombinants that were recombined with no histologically detectable human epithelium in any generation. Upon dissection, there was no definitive approach to histologically confirm the species origin of the recombinant growth. Therefore, due to the amount of labor involved in the tissue recombination assay, serial passage of recombinants was only performed when non-rodent ductal growth was evident upon micro-dissection. Thus, there were instances when recombinants that demonstrated histological human epithelial growth were not recombined and in some occasions recombinants were serially recombined when no human epithelium was detected in the original recombinant; these recombinants were removed from the analysis. In the event where human epithelium was detected in 2^nd^ and/or 3^rd^ generation, human epithelial growth was assumed even if not detected in the histologically analyzed portion in the 1^st^ generation. All recombinant growth was evaluated blinded by a genitourinary pathologist (G.A.), and histological evaluation of all the recombinants was considered benign or hyperplastic with no evidence of cancer. All specimens were analyzed for AMACR expression and only one atrophic gland stained positive (data not shown) but did not represent prostate cancer pathologically.

### *In Vivo* Differentiation of Human Prostate Side Population Recombined with rUGM

**Figure 4:**
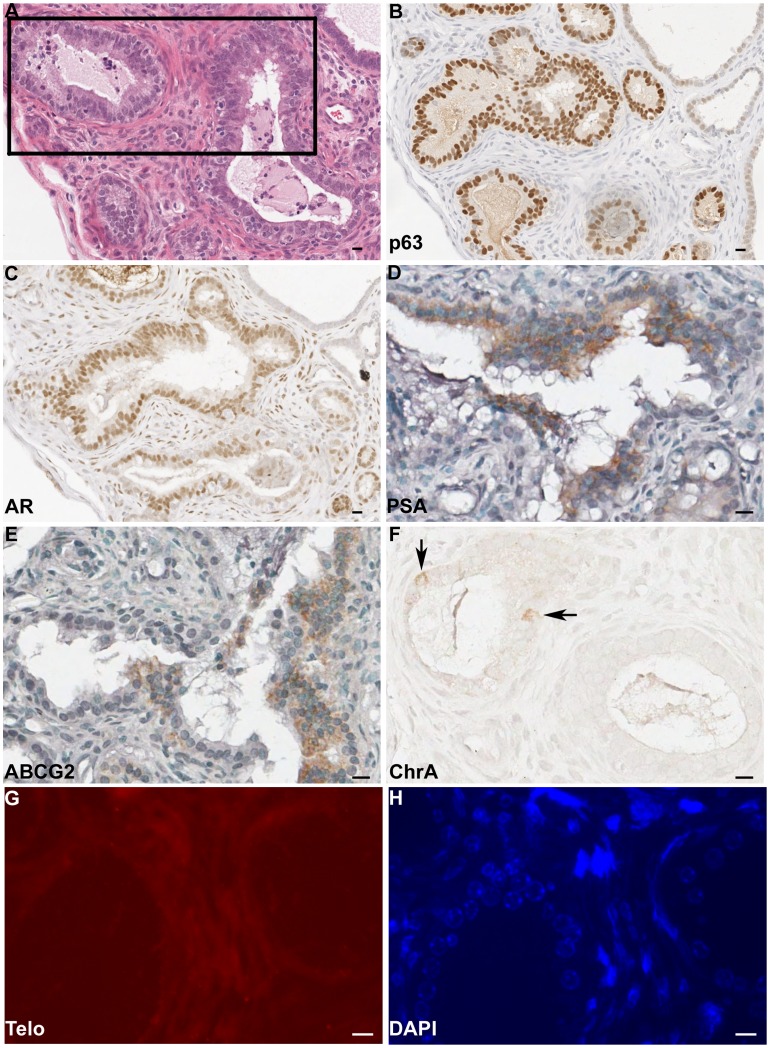
Expression of Differentiation Markers in Recombinants. Nonconsecutive serial sections of a recombinant derived from 250 side population cells from specimen NT6+ rUGM were stained. A) H&E staining, box represents area examined at higher power in D–H; B) p63 IHC; C) AR IHC; D) PSA IHC; E) ABCG2 IHC; F) Chromogranin A (ChrA) IHC arrows indicate positive cells; G) telomere (Telo) detection by FISH; and H) DAPI staining. B-H) Scale bar, 10 µm.

All recombinants containing glands with human epithelial cells were analyzed for markers of differentiation. All glands analyzed for marker expression were negative for the telomere probe (Figure 4G) indicating that they were not of rodent origin and did not demonstrate Hoechst stained punctate nuclei (data not shown) indicating that they were not of mouse origin. When side and non-side population cells were recombined with rUGM, the resulting glands contained epithelial cells from multiple lineages. Most glands contained a continuous p63 expressing basal layer with AR expressing cells (Figure 4B–C). There was no observable difference in the pattern of differentiation between recombinants derived from benign, non-tumor, or tumor specimens nor was the phenotype different between recombinants made with side or non-side populations. In contrast, rodent prostate glands are comprised of a discontinuous p63 positive basal layer. PSA expressing cells were detected in cells in recombinants from side population cells (Figure 4D). PSA is a human specific kallikrein secretory protein which is not expressed in rodent prostate. Detection of PSA expression further demonstrates the human origin of the epithelial compartment and ability of contributing cells to differentiate. Cells expressing chromogranin A were detected indicating the presence of neuroendocrine differentiation (Figure 4F). The presence of multiple epithelial cell lineages through several recombinant generations demonstrates the multi-potentiality of the side population. ABCG2 expressing cells were detected in the recombinants from side population cells (Figures 1A and 4E) indicating the side population was capable of self-renewal. ABCG2 expressing cells in the recombinant also indicate that the stem cell niche has been re-established.

Recombinants from side and non-side population cells isolated from the same patient were compared (Figure 1). Second generation recombinants contained p63 and AR expressing cells. Recombinants with the side population had slightly more intense ABCG2 expression while light PSA expression was only detected in the non-side population recombinant.

## Discussion

The recombination assay demonstrates prostate tissue generation with as low as 50 sorted human prostate cells from freshly digested tissue. The frequency of first or serial generations was not dependent on the number of cells used in recombination (Tables 2 and 3). While fresh undigested human tissue that contains stem cells has been used in tissue recombination as a classic example of this assay [2], most human prostate stem cell studies have focused on using transformed/immortalized cell lines or cells maintained in culture to generate spheres or organoids [9], [28], [29], [30]. The current procedure using sorted human tissue digests recombined with rUGM is extremely labor intensive and requires the coordination of various steps such as tissue acquisition, digestion to single cells, cell sorting, dissection of timed pregnant rodents, and implantation into host animals. These experiments are only possible with a reliable tissue procurement program, flexible FACS capability, and a reliable source of timed pregnant animals. The coordination of these events is a critical step in the identification of human prostate cells with stem cell properties.

The tumorigenic potential of cells isolated from tumor specimens was tested by grafting collagen suspended cells under the renal capsule and in recombination with rUGM. In this study only four radical prostatectomy specimens with areas of histologic tumor large enough to digest, sort, and combine with rUGM was employed for tissue recombination (Table 1). All of the prostatic growth in recombinants containing cells isolated from tumor, non-tumor, or benign tissues was pathologically benign and only observed AMACR expression in one atrophic gland. Furthermore, the control of sorted cells suspended in collagen alone for each recombinant did not grow. The result of no growth from sorted cells grafted alone is not unexpected given the low number of grafts and the historically low xenograft take rate (∼5–10%) of advanced/metastatic tissue from prostate cancer specimens [30]; the zero take rate reported with sorted cells from prostate cancer specimens [31]; and the low grade of tumor specimens used in this study. Earlier studies suggest that tumorigenic prostatic epithelial cells can exhibit a transformed phenotype, but may retain the ability to form normal prostatic structures when associated with an inductive mesenchyme. For example, the Dunning tumor model is tumorigenic when grafted into a male host. However, Dunning tumor recombined with UGM results in grafts that contain areas of tumor and areas of histologically normal prostatic ductal tissues [32]. When Dunning tumor is serially grafted with inductive mesenchyme, tumors do not form and histological analysis of the tissue recombinants demonstrates only normal prostatic structures [33]. Thus, reintroduction of tumor tissue into the inductive and subsequent “normal” stromal environment has the potential to revert a transformed epithelial phenotype back to a histologically “normal” prostatic phenotype [32], [33], [34]. Therefore, a cancer stem cell may have the potential to generate differentiated tumors or differentiated benign tissue depending on the microenvironment, or cancer stem cells were not present in the sorted populations. Toivanen et al., demonstrated that tissue and sorted cells from human prostate cancer specimens recombined with mouse UGM was more likely to give rise to prostate tissue with a prostate cancer phenotype compared to tissue or cells grafted alone [31]. In contrast, we only observed AMACR expression in one atrophic gland and did not observe loss of the p63 layer. Furthermore, no recombination had pathological evidence of prostate cancer. There are several possible explanations for the differing results including but not limited to: We used 50–2000 side population cells in our recombinations compared to 1–5×10^5^ [31] thereby greatly reducing the chance to include a prostate cancer stem cell in the recombinant; the side population may select benign stem cells or basal stem cells; or additional epithelial cells (luminal) are required for cancer stem cell to exhibit a prostate cancer phenotype. There is additional evidence that the number of benign stem cells is probably significantly greater compared to cancer stem cells, or that benign stem cells in prostate tumors are more readily detected using standard stem cell assays. Garraway et al., demonstrated prostaspheres lacked the TMPRSS-ERG fusion when derived from TMPRSS-ERG fusion positive tumors [7] indicating that benign progenitor cells are selected for in the sphere forming assay. Given the rarity of the putative cancer stem cell, enrichment methods based on unique cancer stem cell phenotypes may be required to exclude benign stem cells in order to analyze cancer stem cells.

Tissue recombination with rUGM has been instrumental in the characterization of cells with stem cell properties in the mouse prostate. The mouse provides a powerful model to study prostate stem cells. Mouse prostate cells capable of prostate tissue generation in the tissue recombinant assay usually range in number from 1×10^3^ to 5×10^5^ enriched for Lin^−^Sca^−^1^+^CD133^+^CD44^+^ cells, sphere initiating cells, or cells expressing α_6_ integrin [4], [5], [35]. Notably, a single Lin^−^Sca-1^+^CD133^+^CD44^+^CD117^+^ cell isolated from mouse prostate is capable of prostatic tissue generation in recombination with rUGM in 14/97 grafts [5]. In studies using lineage-tracing in mouse prostate regeneration there are basal stem cells and luminal stem cells that only give rise to the same epithelial lineage [6], it will be interesting to determine if the same is true in human prostate regeneration.

Unlike in the mouse, the use of isolated human prostate cells to perform tissue recombination experiments has been limited due to the low number of cells isolated from specimens procured for research. Vander Griend et al., were the first to establish that 2×10^5^ dissociated primary human prostate epithelial cells could generate prostatic tissue in recombination with rUGM [8]. However FACS isolation of putative stem cells and use in stem cell assays based on CD133 expression was not possible due to the sensitivity of the antibody and the low frequency of CD133 expressing cells [8]. Previously, cells isolated based on CD44 expression and adherent to collagen type IV were cultured and demonstrated prostatic growth in recombination with rUGM [36]. Sphere initiating cells from human prostate specimens were also used to generate prostatic tissue in recombination with rUGM (5×10^3^–5×10^5^ cells were used) [7]. Recently, human prostate cells isolated from tissue based upon Epcam^+^CD44^-^CD49^hi^ cells were more efficient in tubule formation in recombination with human fetal stromal cells when compared to Epcam^+^CD44^+^CD49f^hi^ cells (1×10^5^ were used) [10]. To our knowledge, the only study to use less than 5×10^3^ cells was conducted by using spontaneously immortalized prostate epithelial cells from primary human prostate cells (HPrE) obtained from Cambrex [9]. In these studies 10–5,000 NHPrE1 cells generated prostatic tissue in ∼ 1/3 of recombinants containing rUGM and 5×10^5^ or more NHPrE1 cells generated prostatic tissue in >60% of recombinants. [9] Because of patient variability and the inability to access many surgical specimens within hours of surgical removal, the number of viable cells available for a given experiment ranged between 3.7×10^4^–2.2×10^6^ and the number of viable cells/gram tissue ranged from 9.25×10^3^ to 1.44×10^6^ before isolation of the side population (Table 1). In the current study, as few as 50 side population cells isolated from human specimens in recombination with rUGM resulted in prostatic tissue generation and 125 side population cells resulted in ductal growth in multiple generations. Although not statistically analyzed due to the low number of recombinants and specimen variability, the number of cells used in recombination did not seem to correlate with the frequency of growth in all generations (Tables 2 and 3).

There is an increase in the number of side population recombinants with first generation growth compared to non-side population recombinants with equal number of cells. While this increased frequency of growth may not qualify as enrichment of stem cells in the side population, these results demonstrate that stem cells are present in the side population. The serial regeneration was performed using an indirect assessment of stem cells within the glands that relied on the visual appearance of the pattern of ductal growth as rodent or human. The growth rate of serial generations may have been compromised, because the pattern of ductal growth to distinguish between rodent and human is less robust than molecular markers, such as FISH. The ideal method to generate serial generations would be to reisolate the sorted population from the recombinants to use in serial generations. Unfortunately the size of the recombinants (2–62 µg) prevents the feasibility of this experiment. However, the growth of recombinants with non-side population cells may suggest the presence of stem cells or even refute the idea that only “stem” cells can generate prostate tissue in these assays. The lack of a difference in ductal growth or number of glands per recombinant using representative numbers of side and non-side populations (Figure 3C and 3D) indicates there are rare stem cells in the non-side population. In fact, ABCG2 expressing cells may be present in the non-side population due to sorting inefficiency, resulting in the ABCG2 expressing cells in rare recombinants from non-side population cells (Figure 1B). Alternatively, a stem cell negative for ABCG2 expression may produce ABCG2 expressing cells in this assay. Importantly, cell(s) that can generate a prostate in development, regeneration, and in tissue recombination may not all be equal. Interestingly, the analysis of growth rate of the recombinants demonstrated a high variability of recombinant growth between patients. Cells isolated based on the side population produced at least 1 viable recombinant for each of the seven specimens, while only 3 out of 7 specimens demonstrated at least 1 recombinant growth when an equal number of non-side population cells were used (Table 2). The fact that epithelial cells with multiple lineages are present for several generations demonstrates that cells with multipotency were driving recombinant growth in these assays (Figures 1 and 4). Additionally, recombinants from side population cells contained ABCG2 expressing cells demonstrating self-renewing capabilities and the presence of PSA, chromogranin A, AR, and p63 expressing cells demonstrate multipotency. The areas expressing p63, AR, PSA and ABCG2 sometimes overlapped (Figures 1 and 4B–E) these areas possibly represent a mix of differentiated and primitive cells or intermediate cells that express multiple markers of differentiation. There are conflicting reports on ABCG2 expressing cells in the basal epithelial layer of the prostate. We identified ABCG2 expressing cells have been found within the basal compartment, but failed to find cells that co-expressed both basal markers and ABCG2 [21]. In contrast, Pascal et al., identified ABCG2 cells that either co-expressed basal or endothelial cell markers [20]. Further analysis is required to determine if the ABCG2, p63, AR, PSA expressing cells have multipotency potential or if the phenotype is a result of the tissue recombination assay.

Our data and other studies using cells isolated from human prostate specimens demonstrate a low rate of recombination efficiency. Such studies utilized standard immunocompromised host mice for renal capsule grafting, NOD/SCID mice [7], [9] or athymic nude host [8] and recently NOD/SCID interleukin-2 receptor gamma chain null (Il2rg(−/−)) mice [10]. Perhaps, a more immunocomprimised host would improve recombination efficiency in these studies as was seen in tumorigenicity studies using the NOD/SCID (Il2rg(−/−)) mice [37]. The percentage of recombinants with contributing mouse epithelial cells to ductal growth was 37%. High levels of infiltrating mouse cells dictates the necessity for screening of epithelial species of origin in all recombination experiments [28]. The determination of whether the degree of immune deficiency alters the amount of mouse contribution would be interesting.

These studies demonstrate the ability to generate prostatic growth in the rUGM tissue recombination assay using a low number of sorted cells from clinical specimens of human prostate. Side population cells isolated based on the ABCG2-mediated efflux of DCV have stem cell properties. ABCG2 may play an important role in prostate stem cell maintenance since DHT can be effluxed by ABCG2 and inhibition of ABCG2 results in the induction of AR expression [21]. Future studies are needed to determine whether ABCG2 inhibition has the potential to decrease stem cell maintenance by allowing DHT to initiate differentiation, identifying ABCG2 as a new therapeutic target for prostatic diseases related to a deregulated stem cell niche in disease stages such as prostate cancer and benign prostatic hyperplasia.

## Supporting Information

Figure S1 **Positive and negative human prostate controls for IHC staining.** A) p63 IHC; B) no primary antibody (p63) with goat anti-mouse IgG antibody IHC; C) AR IHC; D) no primary antibody (AR) with goat anti-rabbit IgG antibody IHC; E) PSA IHC; F) no primary antibody (PSA) with goat anti-mouse IgG antibody IHC; G) ABCG2 IHC; H) no primary antibody (ABCG2) with goat anti-mouse IgG antibody IHC; I) Chromogranin A IHC; J) no primary antibody (Chromogranin A) with goat anti-rabbit IgG antibody IHC; K) AMACR IHC; L) no primary antibody (AMACR) with goat anti-rabbit IgG antibody IHCA-L) Scale bar = 10 µm.
(TIF)

Figure S2 **FACS isolation of the side population in human clinical specimens based upon DCV efflux.** A,C,E,G,I,K,M) The gated viable singlets (not shown) are plotted in a double scatter plot between red (650 nm) and blue (450/40 nm) emission and are isolated based upon efflux of DCV. B,D,F,H,J,L,N) ABCG2-mediated DCV efflux is inhibited in the presence of FTC to establish where the side population (SP) and non-side population (NSP) gates should be placed. Purple: Viable cells; Green: NSP; Blue: SP.
(TIF)

Figure S3 **Recombinants with rodent epithelium.** A) Rodent ductal growth in observed in micro-dissection and H&E analysis. B) Infiltrating mouse epithelium observed in H&E and Hoechst (not shown) analysis, but not observed in micro-dissection. Ruler scale = mm; Scale bar = 50 µm
(TIF).

Figure S4 **Positive and negative controls for rodent telomere FISH analysis and mouse cell detection with Hoechst.** Mouse control tissue A) Telomere FISH analysis positive for telomere repeats; B) DAPI counter stain same field; C) Hoechst stain demonstrating punctate nuclei in mouse tissue. Human control tissue D) Telomere FISH analysis negative for telomere repeats; E) DAPI counter stain same field; F) Hoechst stain demonstrating non-punctate nuclei. A-F) Scale bar = 10 µm.
(TIF)
