# Supplementary figures and images for: Human Prostate Side Population Cells Demonstrate Stem Cell Properties in Recombination with Urogenital Sinus Mesenchyme
Source: PLoS One. 2013 Jan 31;8(1):e55062. doi: 10.1371/journal.pone.0055062 (PMC3561453; doi:10.1371/journal.pone.0055062)

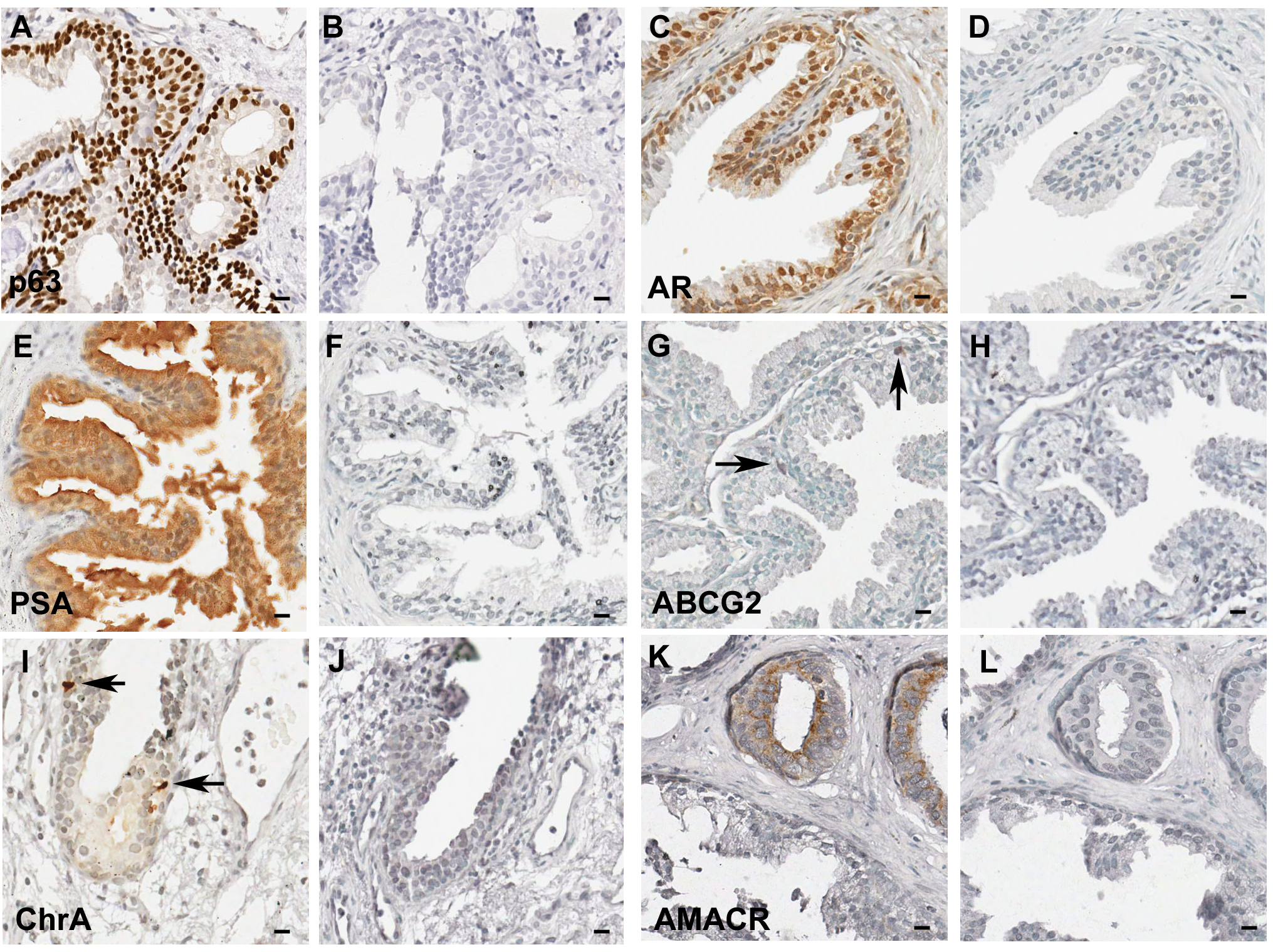

Supplement: Figure S1 — Positive and negative human prostate controls for IHC staining. A) p63 IHC; B) no primary antibody (p63) with goat anti-mouse IgG antibody IHC; C) AR IHC; D) no primary antibody (AR) with goat anti-rabbit IgG antibody IHC; E) PSA IHC; F) no primary antibody (PSA) with goat anti-mouse IgG antibody IHC; G) ABCG2 IHC; H) no primary antibody (ABCG2) with goat anti-mouse IgG antibody IHC; I) Chromogranin A IHC; J) no primary antibody (Chromogranin A) with goat anti-rabbit IgG antibody IHC; K) AMACR IHC; L) no primary antibody (AMACR) with goat anti-rabbit IgG antibody IHCA-L) Scale bar = 10 µm. (TIF) [file pone.0055062.s001.tif]

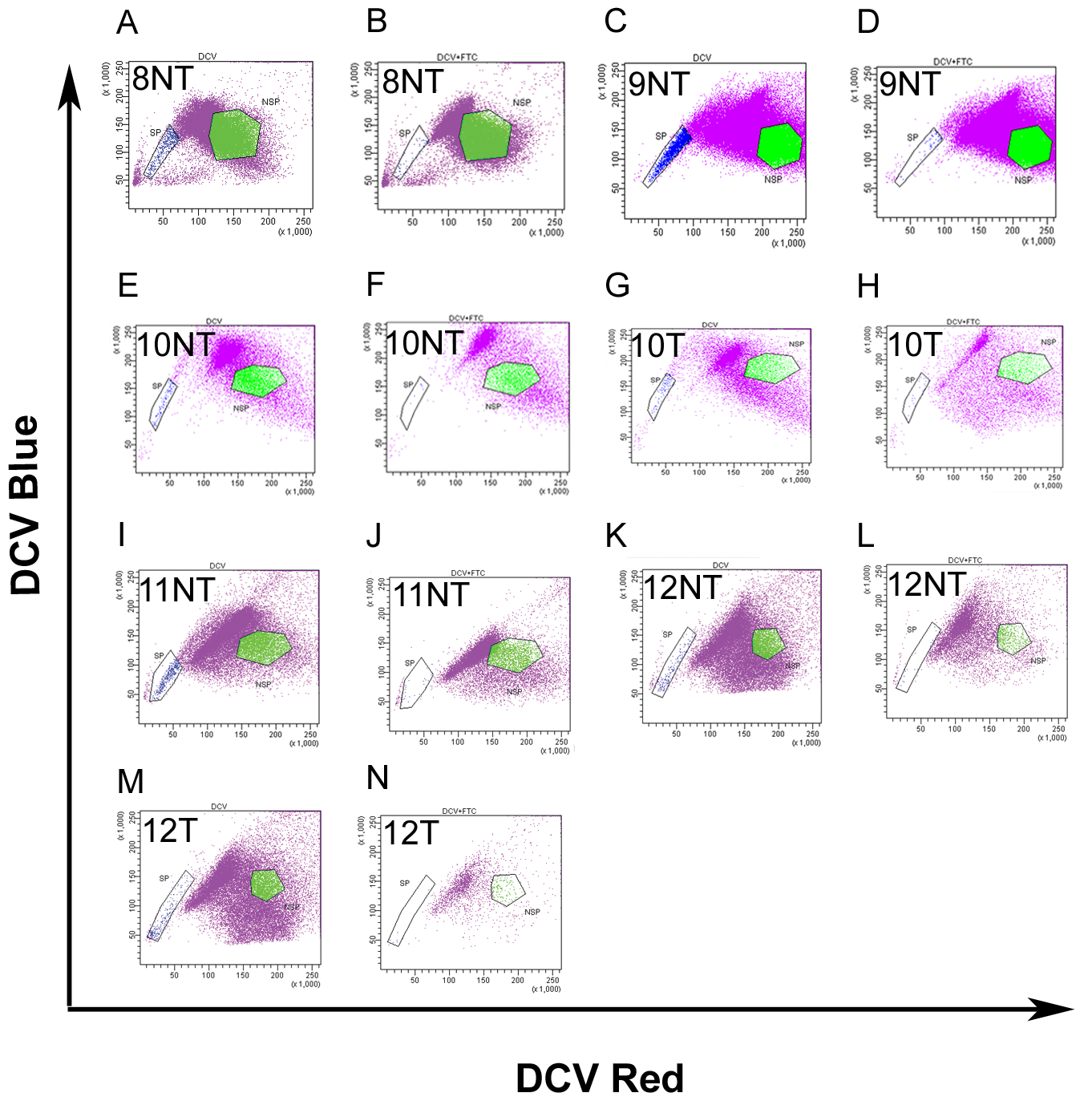

Supplement: Figure S2 — FACS isolation of the side population in human clinical specimens based upon DCV efflux. A,C,E,G,I,K,M) The gated viable singlets (not shown) are plotted in a double scatter plot between red (650 nm) and blue (450/40 nm) emission and are isolated based upon efflux of DCV. B,D,F,H,J,L,N) ABCG2-mediated DCV efflux is inhibited in the presence of FTC to establish where the side population (SP) and non-side population (NSP) gates should be placed. Purple: Viable cells; Green: NSP; Blue: SP. (TIF) [file pone.0055062.s002.tif]

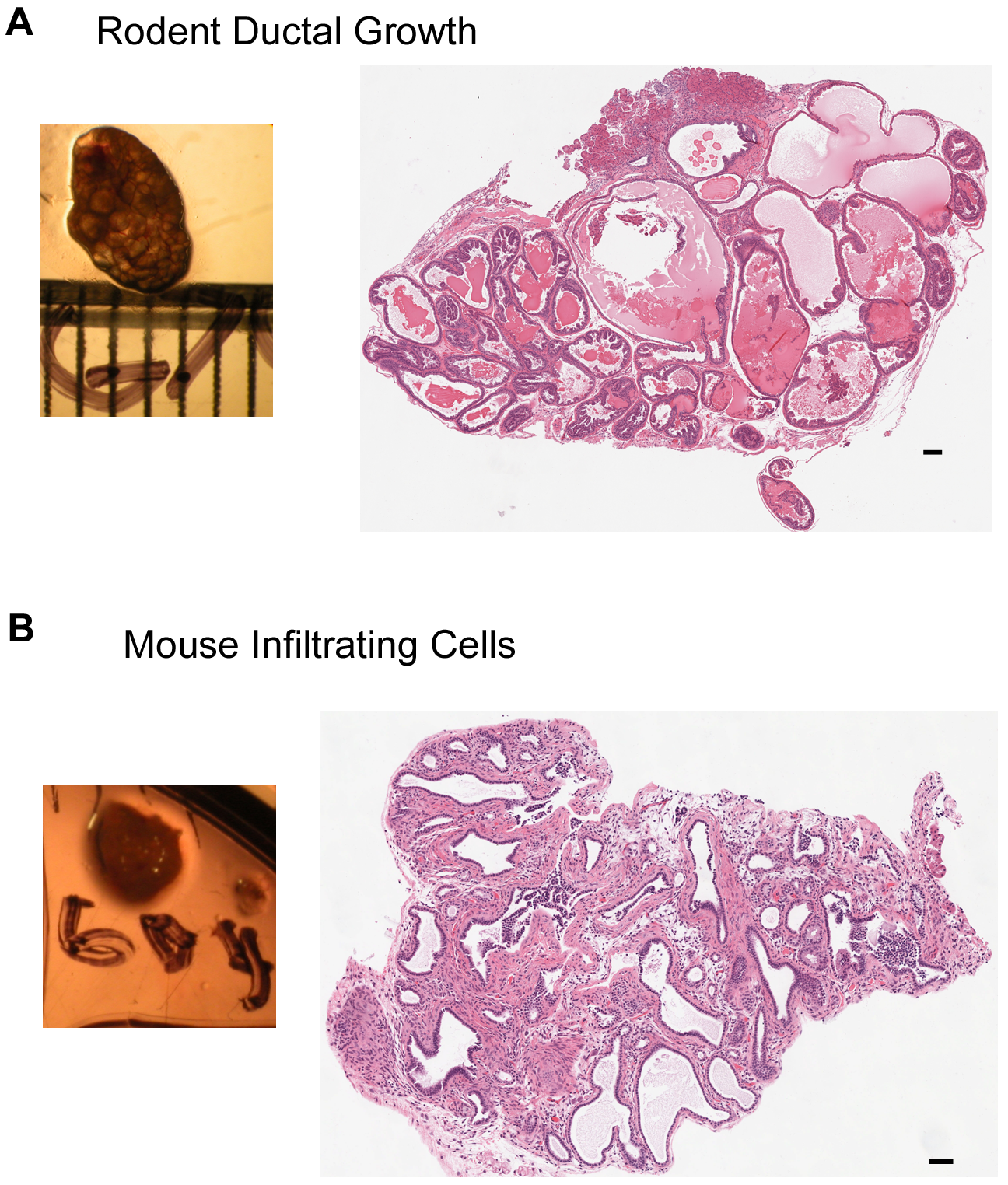

Supplement: Figure S3 — Recombinants with rodent epithelium. A) Rodent ductal growth in observed in micro-dissection and H&E analysis. B) Infiltrating mouse epithelium observed in H&E and Hoechst (not shown) analysis, but not observed in micro-dissection. Ruler scale = mm; Scale bar = 50 µm (TIF). [file pone.0055062.s003.tif]

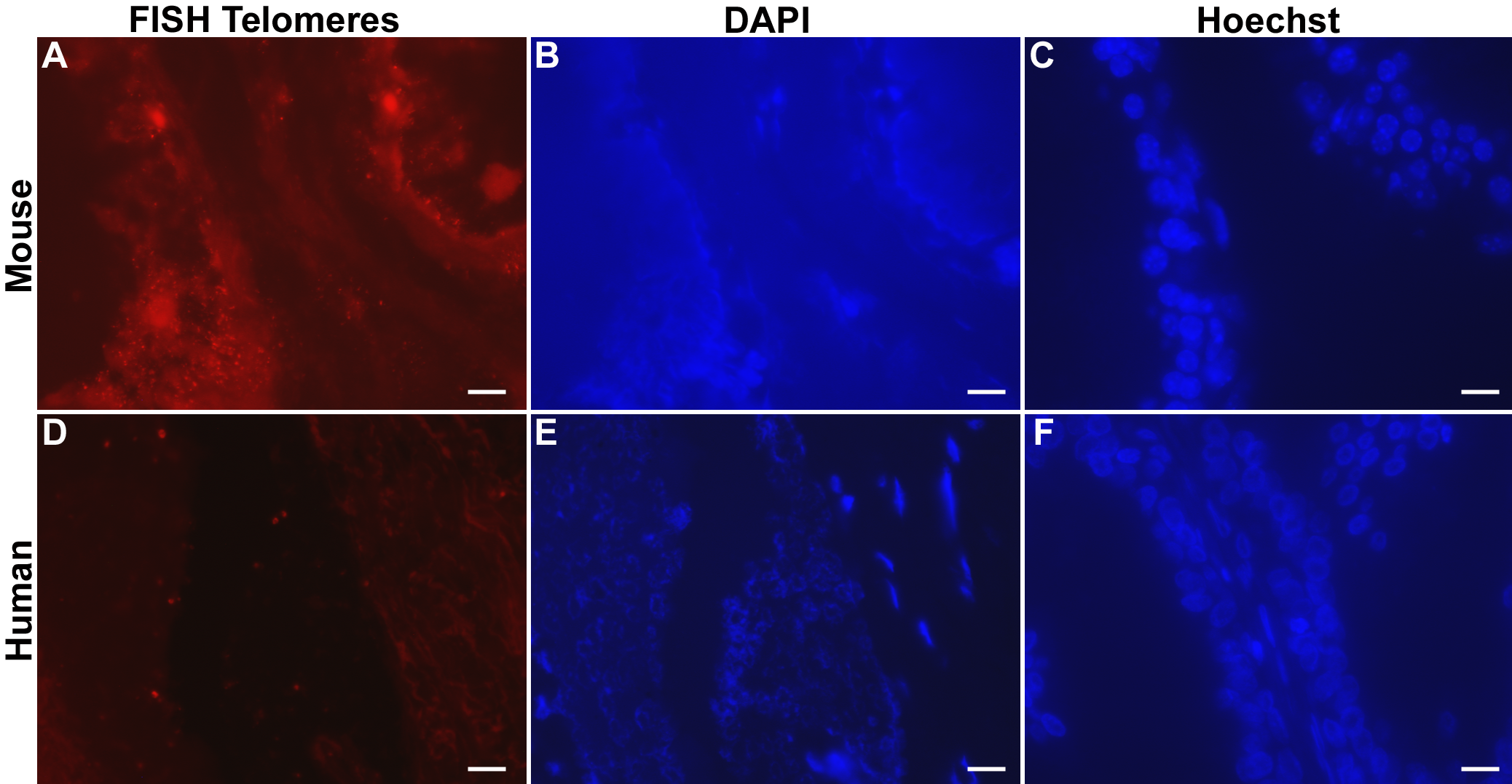

Supplement: Figure S4 — Positive and negative controls for rodent telomere FISH analysis and mouse cell detection with Hoechst. Mouse control tissue A) Telomere FISH analysis positive for telomere repeats; B) DAPI counter stain same field; C) Hoechst stain demonstrating punctate nuclei in mouse tissue. Human control tissue D) Telomere FISH analysis negative for telomere repeats; E) DAPI counter stain same field; F) Hoechst stain demonstrating non-punctate nuclei. A-F) Scale bar = 10 µm. (TIF) [file pone.0055062.s004.tif]
